# Circulating microRNA Expression in Cushing’s Syndrome

**DOI:** 10.3389/fendo.2021.620012

**Published:** 2021-02-22

**Authors:** Sharmilee Vetrivel, Ru Zhang, Mareen Engel, Barbara Altieri, Leah Braun, Andrea Osswald, Martin Bidlingmaier, Martin Fassnacht, Felix Beuschlein, Martin Reincke, Alon Chen, Silviu Sbiera, Anna Riester

**Affiliations:** ^1^Department of Endocrinology, Medizinische Klinik und Poliklinik IV, Ludwig-Maximilians-University, Munich, Germany; ^2^Department of Stress Neurobiology and Neurogenetics, Max Planck Institute of Psychiatry, Munich, Germany; ^3^Division of Endocrinology and Diabetes, Department of Internal Medicine I, University Hospital, University of Würzburg, Würzburg, Germany; ^4^Klinik für Endokrinologie, Diabetologie und Klinische Ernährung, Universitätsspital Zürich, Zürich, Switzerland; ^5^Department of Neurobiology, Weizmann Institute of Science, Rehovot, Israel

**Keywords:** cortisol, ACTH, miRNA, biomarker, cortisol-producing adenoma, miR-182-5p, hypercortisolism, miR-183 cluster

## Abstract

**Context:**

Cushing’s syndrome (CS) is a rare disease of endogenous hypercortisolism associated with high morbidity and mortality. Diagnosis and classification of CS is still challenging.

**Objective:**

Circulating microRNAs (miRNAs) are minimally invasive diagnostic markers. Our aim was to characterize the circulating miRNA profiles of CS patients and to identify distinct profiles between the two major CS subtypes.

**Methods:**

We included three groups of patients from the German Cushing’s registry: ACTH-independent CS (Cortisol-Producing-Adenoma; CPA), ACTH-dependent pituitary CS (Cushing’s Disease; CD), and patients in whom CS had been ruled out (controls). Profiling of miRNAs was performed by next-generation-sequencing (NGS) in serum samples of 15 CS patients (each before and after curative surgery) and 10 controls. Significant miRNAs were first validated by qPCR in the discovery cohort and then in an independent validation cohort of 20 CS patients and 11 controls.

**Results:**

NGS identified 411 circulating miRNAs. Differential expression of 14 miRNAs were found in the pre- and postoperative groups. qPCR in the discovery cohort validated 5 of the significant miRNAs from the preoperative group analyses. Only, miR-182-5p was found to be significantly upregulated in the CD group of the validation cohort. Comparing all CS samples as a group with the controls did not reveal any significant differences in expression.

**Outcome:**

In conclusion, our study identified miR-182-5p as a possible biomarker for CD, which has to be validated in a prospective cohort. Furthermore, our results suggest that presence or absence of ACTH might be at least as relevant for miRNA expression as hypercortisolism itself.

## Introduction

Cushing’s syndrome (CS) is a severe disease resulting from prolonged exposure to excessively high levels of cortisol (1). In the majority of patients hypercortisolism is due to ACTH secretion by corticotroph adenomas of the pituitary gland resulting in Cushing’s disease (CD) (2). In approximately 20% of cases cortisol is secreted autonomously by the adrenal cortex. Adrenal dependent CS is mostly caused by unilateral cortisol-producing adrenal adenomas (CPA), rare causes are cortisol-secreting adrenocortical carcinomas (ACC), primary bilateral macronodular adrenocortical hyperplasia (PBMAH), bilateral CPAs and primary pigmented micronodular adrenal disease (PPMAD) (3, 4). Prolonged exposure to cortisol causes visceral obesity, resistance to insulin, osteoporosis, altered lipid and glucose metabolism, hypercoagulability, neuropsychiatric disorders and hypertension (5). The clinical features of CS vary widely, and no single specific symptom is present in every patient, making the diagnosis and subtyping of CS difficult and subject to false-positive and false-negative test results (6). However, timely and precise diagnosis of CS is crucial to avoid the high mortality and morbidity of affected patients (7). This clinical situation calls for more reliable, specific, and selective biomarkers for diagnosing CS.

MicroRNAs (miRNAs) are short (20–24 nucleotides) non-coding RNA molecules with diverse cellular regulatory functions including differentiation, proliferation and apoptosis (8). While the majority of miRNAs exist intracellularly, a significant number of miRNAs have been observed in extracellular compartments including plasma, serum, urine, saliva, semen, ascites, amniotic pleural effusions, and cerebrospinal fluid (9, 10). There are two major populations of circulating miRNAs, those vesicle-associated (which represents 90% of circulating miRNAs) and those non–vesicle-associated (11). Circulating miRNA profiles have been found to be very dynamic and abnormal levels of distinct miRNAs could be observed in specific biological stages of diseases and particularly during the development, invasion and metastasis of cancer (12). Circulating miRNAs fulfil several properties of non-invasive biomarkers, such as availability in various bodily fluids, sequence conservation between human and various preclinical models and availability of sensitive technologies for their quantification (13).

In recent years circulating miRNAs have been investigated as potential biomarkers for adrenal diseases with a total of eight studies investigating the pattern of extracellular miRNAs in adrenocortical tumors to date (14). The miRNAs investigated were found to aid in different forms of adrenal adenomas (15). Importantly, circulating miRNAs, miR-34a, and miR-483-5p were identified as candidate serum biomarkers distinguishing between benign and malignant adrenocortical tumors (16). In ectopic ACTH-dependent CS, there has been a single study reporting differences in plasma miR-expression (17). However, to the best of our knowledge, studies comparing different types of CS with each other have not yet been performed. The aim of this study was to compare circulating miRNA expression profiles of patients with different forms of CS and controls and evaluate their applicability as biomarkers. Therefore, we compared circulating miRNA profiles in serum samples of patients with CPA and patients with CD with those of patients in whom CS had been ruled out (controls). In addition to preoperative serum samples, we have also analyzed miRNA profiles in paired pre- and post-operative samples. To investigate possible short-term changes in miRNA profiles, we also analyzed serum samples after 1 mg low-dose dexamethasone testing in control patients.

## Materials and Methods

### Patients and Ethics Approval

We conducted a retrospective, bi-centric study, based on a collection of pre- and post-operative serum samples of patients with Cushing’s syndrome and controls at the Endocrinology Unit of the University Hospital of Munich and of the University Hospital of Würzburg. Diagnosis of CS followed current guidelines was based on the presence of relevant clinical features and biochemical confirmation through the following screening tests: increased 24-hour urinary free cortisol; loss of diurnal circadian cortisol rhythm with midnight salivary or serum cortisol concentrations; insufficiently suppressed serum cortisol levels after overnight administration of 1 mg of dexamethasone and suppressed plasma ACTH levels (as shown in **Table 1**). All patients were registered as part of ongoing registries and biobanks (ENS@T, European Network for the Study of Adrenal Tumor; NeoExNet, Exzellenz-Netzwerkes für neuroendokrine Tumoren Muenchen). The study was approved by the Ethics Committees of the University of Munich and Würzburg and written informed consent was obtained from all enrolled patients. All experiments were performed according to current guidelines and protocols. The discovery cohort contained 5 patients with overt adrenal dependent CS (CPA), 10 patients with overt pituitary dependent CS scheduled for surgery (CD) and 10 patients who were submitted in our outpatient clinic with suspicion of CS and in whom CS was ruled out (controls). Serum of the patients with CPA and CD was collected at the time of diagnosis preoperatively and after successful adrenalectomy and transsphenoidal pituitary surgery, respectively. Therefore, in total 40 serum samples of the discovery cohort were used for next-generation-sequencing (NGS): 5 preoperative CPA, 5 postoperative CPA, 10 preoperative CD, 10 postoperative CD, and 10 controls. As a confirmatory cohort, an independent series of 11 patients with CPA, 9 patients with CD and 11 controls were analyzed. Of the 11 controls of the confirmatory cohort also serum samples after 1 mg dexamethasone test were included (**Table 1**). Age and sex did not differ significantly between the groups (**Table 1**). All blood samples were collected in the morning (08:00–11:00 AM). All of our patients with CPA and CD, respectively, revealed postoperative adrenal insufficiency and were in need of a cortisol replacement therapy. At the timepoint of the postoperative blood sampling 87% of the patient still received hydrocortisone between 10 to 25 mg.

**Table 1:** Clinical characteristics of the patient groups.

		Age at diagnosis/surgery [years]	Sex [%Female]	BMI [kg/m²]	Hypertension[%positive]	Diabetes [% positive]	baseline ACTH [pg/ml]	Cortisol 24h Urine [nmol/24h]	midnight Cortisol[nmol/l]	Cortisol after 1 mg Dexamethasone[nmol/l]
Discovery Cohort	Controls (n=10)	50 [43;56]	50	33 [30:35]	70	30	3.1 [2.7;3.7]	549 [375;610]	24.8 [17.4;42.2]	27.6 [22.1;29.7]
	CPA (n=5)	58 [48;63]	60	27 [24;28]	60	40	0.4 [0.4;0.4]	876 [506;1346]	242.9 [135.2;397.4]	347.8 [135.2;408.5]
	CD (n=10)	51 [40;57]	50	31 [26;33]	80	50	16.7 [11.0;21.4]	2122 [1991;2868]	165.6 [74.5;460.9]	425.0 [339.5;778.3]
Validation Cohort	Controls (n=11)	45 [29;52]	36	36 [31;40]	54	27	2.2 [2.0;2.9]	270 [223;345]	41.4 [35.9;49.7]	30.4 [24.8;41.4]
	CPA (n=11)	50 [43;53]	72	29 [26;36]	81	36	1.9 [1.6;2.0]	639 [318; 946]	276.0 [182.2;350.5]	532.7 [386.4;623.8]
	CD (n=9)	47 [41;58]	88	26 [25;41]	66	44	14.0 [7.2;21.1]	243 [152; 314]	160.1 [91.1;34.0]	201.5 [121.4;634.8]

Data are given as median with 25th and 75th percentile in brackets.

CPA, cortisol producing adenoma; CD, Cushing’s disease; BMI, body mass index.

### RNA Extraction

Total RNA isolation was carried out from all serum samples (450 µl) by miRNeasy Serum/Plasma Kit (Qiagen GmbH, Hilden, Germany) and stored at −80°C until further use.

### Sample Processing and miRNA Expression Profiling from Serum Samples by Next-Generation Sequencing (NGS)

RNA integrity and absence of DNA was confirmed by Bioanalyzer RNA Nano chips (Agilent Technologies, St. Louis, MO) and Qubit DNA High sensitivity kit, respectively. Sequencing libraries were prepared using the Illumina TruSeq Small RNA Library Preparation Kit. Next generation sequencing was performed on 2 lanes of an Illumina HiSeq2500 (Illumina, San Diego, CA) multiplexing all samples (single end read, 50 bp). The quality of sequencing reads was verified using FastQC0.11.5 (http://www.bioinformatics.babraham.ac.uk/projects/fastqc) before and after trimming. Adapters were trimmed using cutadapt (18). Reads with <15 bp and >40 bp insert sequences were discarded. Alignment of reads was performed using miRBase V21 (19) with sRNAbench (20). For normalization and identification of differentially expressed miRNAs EdgeR and DeSeq in R was used (21–23). miRNAs with an at least 5 raw count per library were included. Disease groups were compared using the unpaired Mann–Whitney test, and, to decrease the false discovery rate, a corrected p-value was calculated using the Benjamini–Hochberg method. Adjusted p < 0.05 and log2 fold of change >1.5 were the cut-off for significance. The RNA seq data generated in this study have been submitted to the NCBI GEO with accession number GSE156693.

### Validation of Individual miRNAs

Significantly differentially expressed miRNAs found by NGS were validated by RT-qPCR. Reverse transcription of RNA was performed using the TaqMan MicroRNA Reverse Transcription Kit (Thermo Fisher Scientific). As reference miRNA, hsa-miR-16-5p was used (12, 24, 25). Quantitative real-time PCR was performed using the TaqMan Fast Universal PCR Master Mix (2x) (CN: 4352042; Thermo Fisher Scientific) on a Quantstudio 7 Flex Real-Time PCR System (Thermo Fisher Scientific) in accordance with the manufacturer’s protocol for TaqMan Advanced miRNA assays (CN: A25576; Thermo Fisher Scientific). The miRNAs along with their respective TaqMan assay IDs are given in **Table S1** **(**26). Contamination controls contained no cDNA templates.

### Statistical Analysis and Software

R version 3.6.1 was used for statistical analyses. To identify miRNAs differentially expressed, generalized linear model (GLM, a flexible generalization of ordinary linear regression that allows for variables that have distribution patterns other than a normal distribution) in the software package edgeR (Empirical Analysis of DGE in R) was employed to calculate *p*-values (25, 27). *p*-values were adjusted using the Benjamini–Hochberg false discovery rate (FDR) procedure (27). GraphPad Prism Version 8 was used for statistical analysis of qPCR results. To quantify the miRNAs in qPCR, the dCt method [delta Ct (cycle threshold) value equals target miRNA’s Ct minus housekeeping miRNA’s Ct was used (Excel 2016, Microsoft, Redmond, WA, USA). The ANOVA test with Bonferroni correction for three groups and Mann-Whitney test for two groups (CS vs Controls) were used for differentially expressed comparisons (28, 29). Receiver operating characteristic (ROC) analysis was performed on miRNAs that could have potential utility as minimally invasive biomarkers. p < 0.05 was considered significant.

## Results

### miRNA Expression Profiles

**Figure 1:**
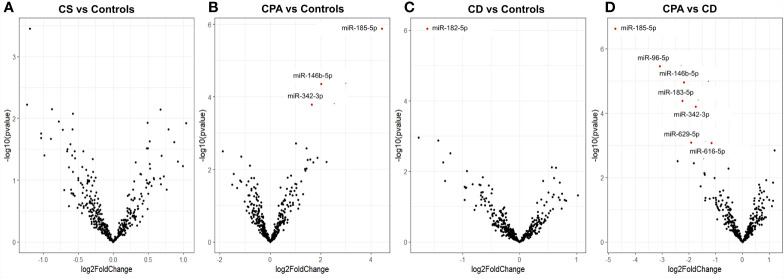
Differentially expressed miRNAs from NGS of preoperative group. Volcano plot showing the relationship between fold change (log2foldchange) and statistical significance (−log10pvalue). The red points in the plot represent the differentially expressed miRNAs with FDR <0.05 considered as statistically significant. CPA, cortisol producing adenoma; CD, Cushing’s Disease, CS, Cushing syndrome represents CPA and CD, taken together. Volcano plot showing the relationship between fold change (log2foldchange) and statistical significance (-log10pvalue) for the groups CS vs Controls **(A)**, CPA vs. Controls **(B)**, CD vs. Controls **(C)**, CPA vs CD **(D)**.

NGS was performed in the discovery cohort of 5 CPA (before and after curative surgery), 10 CD (before and after curative surgery), and 10 control serum samples (total n= 40) and detected a total of 411 miRNAs. No significant influence of batch preparation, age, and sex on the miRNA expression profile was found [**Figure S1** (26)]. Differential analysis was performed between the groups (CPA, CD, and controls) to identify any characteristic miRNA profile associated with one of the entities before and after surgery, respectively. No significant changes were observed based on the comparison of all CS samples pooled together versus control samples (**Figure 1A**). Differential expression levels in three miRNAs, namely miR-185-5p, miR-146b-5p, and miR-342-3p were found when comparing CPA samples with controls (**Figure 1B**), while only miR-182-5p was found to be significantly regulated between CD samples and controls (**Figure 1C**). Post-hoc analyses between CPA and CD revealed that the most pronounced differences included 6 miRNAs: miR-96-5p, miR-146b-5p, miR-183-5p, miR-185-5p, miR-616-5p, and miR-629-5p (**Figure 1D**). Sequencing of the postoperative samples showed significantly different expression of four miRNAs (miR-429, miR-141-3p, miR-215-5p, miR-200a-3p) between CPA and controls [**Figure S2B** (26)]. No significant differences were observed while comparing all postoperative CS samples pooled together with control samples [**Figure S2A** (26)], the postoperative CD samples with controls [**Figure S2C** (26)] and the postoperative CPA serum samples vs CD postoperative samples [**Figure S2D** (26)]. Analysis of the preoperative samples revealed no major difference in the distribution of the top 20 abundant genes compared to the controls [**Figure S3** (26)].

### Validation of Selected miRNAs by qPCR

**Figure 2:**
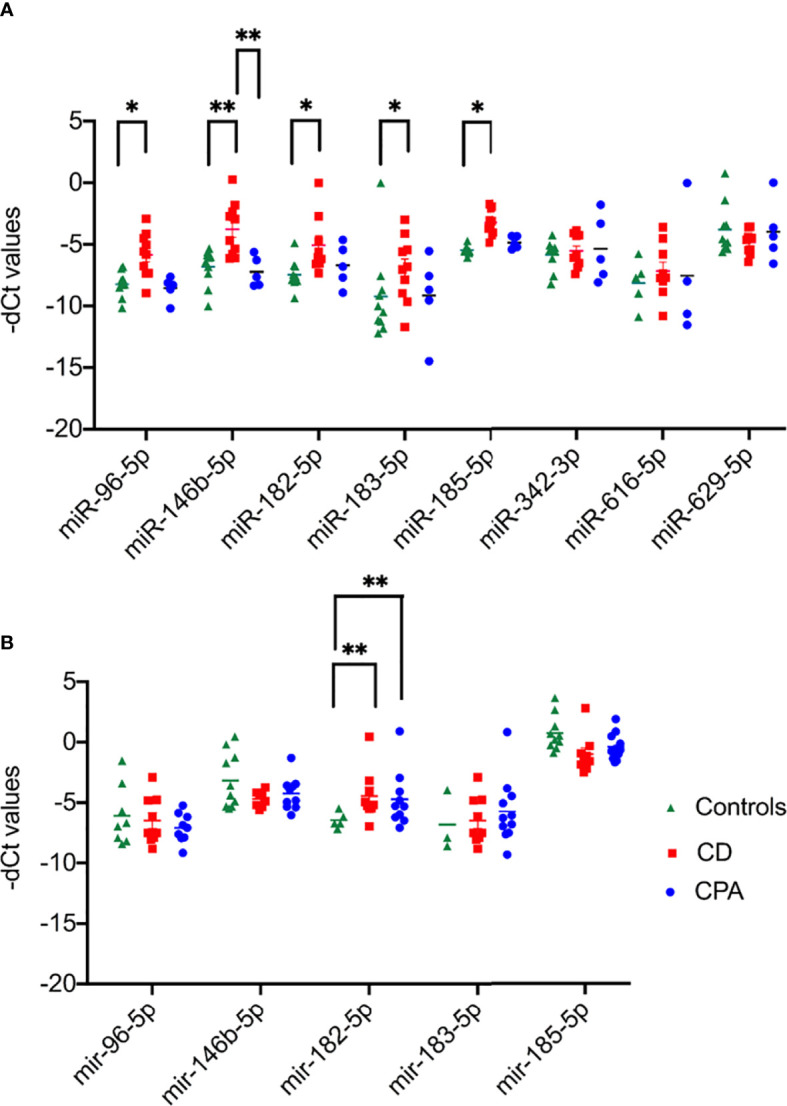
Results of qPCR of the significant miRNAs of preoperative group. Data are represented as Mean ± Standard deviation (SD) of -dCT values. **(A)** Expression analysis in discovery cohort selected for validation from NGS. **(B)** Expression analysis in validation cohort of the significant miRNAs from discovery cohort. Statistics: ANOVA test with Bonferroni correction to detect significant differences between patient groups with at least a signification of p-value < 0.05. *P < 0.05 and **P < 0.01.

**Figure 3:**
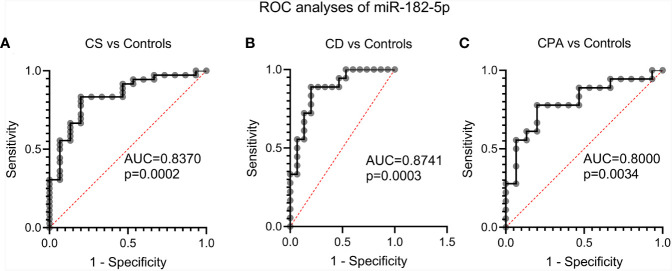
Evaluation of the diagnostic applicability of miR-182-5p by receiver operating characteristic (ROC) curves. ROC curves were plotted for CS (represents CPA and CD) vs controls **(A)**, CD vs. controls **(B)** and CPA vs. controls **(C)** from the discovery and validation cohort. CPA, cortisol producing adenoma; CD, Cushing’s Disease, CS, Cushing syndrome represents CPA and CD, taken together.

**Figure 4:**
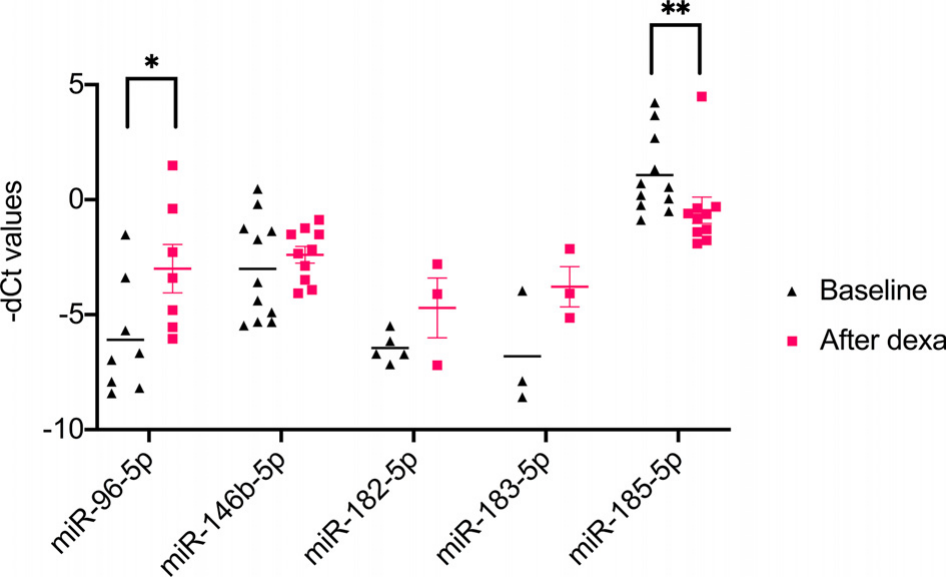
Results of qPCR analysis of 1 mg dexamethasone test on circulating miRNA. Data are represented as Mean ± Standard deviation (SD) of -dCT values Statistics: ANOVA test with Bonferroni correction to detect significant differences between patient groups with at least a signification of p value <0.05. *P<0.05 and **P<0.01.

NGS identified 8 miRNAs that were significantly dysregulated in the preoperative group and 4 miRNAs that were significantly altered in the postoperative group (see **Table 2**). qPCR analysis of these miRNAs revealed significant differences in 5 out of the 8 preoperative miRNAs (miR-185-5p, miR-183-5p, miR-182-5p, miR-146b-5p, and miR-96-5p), but in none of the postoperative cohort samples [**Figure S4** (26)]. To further validate the results from the discovery cohort, we assessed the remaining five significant miRNAs in an independent set of samples (CPA, n=11; CD, n=9 controls, n=11). Only miR-182-5p was confirmed to be significantly upregulated in the preoperative group of the independent cohort (**Figure 2B**). The diagnostic utility of miR-182-5p was evaluated using a ROC analysis (**Figure 3**). For CD samples from both discovery and validation cohorts, the area under curve (AUC) was 0.87, (95% confidence interval: 0.7 to 1.0, P = 0.0003), while for the CS samples pooled together the area under curve (AUC) was 0.84 (95% confidence interval:0.7 to 0.9, P = 0.0002). The AUC was further lower for CPA samples, 0.8 (95% confidence interval:0.5 to 0.9, P = 0.003). Further, correlation analyses of the gene expression levels with clinical parameters revealed no significant results (**Table S2**). As none of the postoperative miRNAs were found to be significantly different in the discovery cohort, they were not further analyzed in the validation cohort. Finally, to investigate whether the five selected circulating miRNAs are affected by short-term exogenous glucocorticoids, we analyzed the expression levels of the miRNAs in serum samples before and after 1 mg of dexamethasone was given 9 h before the control patients of the independent cohort. miR-185-5p and miR-96-5p expression was found to be significantly modulated by dexamethasone treatment (**Figure 4**).

**Table 2:** Comparison of results of RNA Sequencing and qPCR in selected miRNAs.

RNA sequencing						qPCR		
Gene symbol	Mean Count	Position	CPA vs Controls	CD vs Controls	CPA vs CD	CPA vs Controls	CD vs Controls	CPA vs CD
miR-96-5p	689	122	1.31	−1.77	−3.08***	−0.22	1.77*	−1.99*
miR-146b-5p	842	114	2.03*	−0.16	−2.18**	−0.38	2.57**	−2.95*
**miR-182-5p**	**5,493**	**46**	**−0.58**	−1.62***	**−1.03**	**0.75**	1.73*	**−0.98**
miR-183-5p	885	112	0.81	−1.43	−2.23**	0.07	2.20*	−2.14
miR-185-5p	40	313	4.43	−0.31	−4.74***	0.54	1.78*	−1.24
miR-342-3p	1,024	105	1.64	−0.10	−1.75*	0.47	0.31	0.16
miR-616-5p	31	337	0.83	−0.32	−1.15*	0.59	0.99	−0.40
miR-629-5p	37	318	1.59	−0.33	−1.92*	−0.15	−1.02	0.87

*P < 0.05, **P < 0.01, and ***P < 0.001. Gene expression is represented as log2fold change. CPA, cortisol producing adenoma; CD, Cushing’s disease. The potential biomarker candidate has been highlighted in bold.

## Discussion

Diagnosis of CS is still challenging due to the broad symptomatology and the clinical overlap with metabolic syndrome (30). Upon CS diagnosis, treatment is complicated by difficulties in distinguishing between the different subtypes of CS. Of the CS subtypes, CD is the dominant form and a variety of dynamic tests and robust imaging procedures are necessary for diagnosis (5). Unsuspected pituitary and adrenal incidentalomas are increasingly found with the widespread use of abdominal and cranial imaging (31). For pituitary incidentaloma the rate ranges between 3.7% and 37% (32). Therefore, we hypothesized that a potential miRNA based diagnostic should aid in the diagnosis of CS or its subtypes as circulating miRNAs have proven their potential as minimal invasive biomarkers in other diseases (33). Few studies have investigated circulating miRNA profiles in CS, and appropriate controls matched for sex, BMI and comorbidities were missing (17, 34). Therefore, in our study we included clinically relevant control samples to identify a unique circulating miRNA signature associated with CS. The control group consisted of patients submitted with the suspicion of CS in the outpatient clinic but in whom CS was ruled out by several diagnostic tests and follow-up. We decided deliberately for such a control group, as this reflects clinical practice and a new biomarker would have to demonstrate relevance in this clinical scenario. In addition, to ensure technical consistency across the various biological samples, the RNA from different groups, including controls, were isolated in batches and possible batch preparation effect was also analyzed in the NGS data. As can be seen [**Figure S1** (26)], no major changes were found, indicating the changes observed in the NGS data, likely arises from inherent biological differences and not due to batch effect. The sequencing data were also found to not be influenced by both age and sex [**Figure S1** (26)].Only miR-182-5p was confirmed as differentially expressed by qPCR of the discovery and the validation cohorts. ROC analysis for the diagnostic power of miR-182-5p resulted in an AUC of 0.89 in separating CD patients from controls, and an AUC of 0.85 in differentiating all patients with CS from controls. This suggests that circulating miR-182-5p may function as a biomarker specifically for CD. Our observation draws strength from related studies wherein circulating miR-182-5p has been successfully identified as a diagnostic biomarker in colorectal adenocarcinoma (35) and coronary artery disease (36). Furthermore, the overexpression of miR-182-5p in prolactin-secreting pituitary adenomas tissues has also been documented (37). However, the potential use of circulating miR-182-5p as a biomarker in CD should be seen with caution due to the differences observed between NGS and qPCR. While miR-182-5p was found to be significantly downregulated in CD in comparison to controls by NGS (**Table 2**), qPCR in discovery and validation cohorts revealed significant upregulation of CD in comparison to controls (**Table 2** and **Figure 2**). This difference could be possibly explained by the high sensitivity observed with qPCR in comparison to the high throughput genomic profiling technologies including NGS and microarray (38). Concurrently, studies with a wholesome approach to circulating miRNAs, namely through microarray and NGS, are often not able to validate their sequencing and array results (25, 34). miR-182-5p along with miR-96-5p and miR-183-5p, were also found to be deregulated in CPA and CD samples compared to the controls (**Figures 1B, C**). Altogether these miRNas belong to the miR-183 cluster. This miR-183 cluster is known to play a role in a variety of non-sensory diseases, including cancer and neurological, psychiatric and auto-immune disorders (39). Furthermore, the expression profiles of the miR-183 cluster was found to be altered in adrenal tissues of patients with pheochromocytoma (40).

Surprisingly, NGS did not identify any distinct miRNA expression pattern between CS taken as a single group and the control group (**Figure 1A**). ACTH itself might lead to changes in the circulating miRNA profile that are more relevant as the hypercortisolism which is present in both forms of CS. The modulation of miRNAs by ACTH has been characterized *in mice* (41) but distinct studies of ACTH modulation on circulating miRNAs are missing. We speculate that the similarity in phenotype between CS and this specific control group might have contributed to this observation, too. Additionally, including only patients with benign tumors might have led to the discovery of a low amount of changes in circulating miRNA profiles as cell disruption and release of miRNAs from tumour cells into the blood, typical features of malignant neoplasms, are minimal.

The CPA samples show a far more diverging pattern for their qPCR expression patterns in both the validation and discovery cohort. While only miRNA-146b and miRNA-96-5p were found to be significantly altered between CPA and controls of the discovery cohort, similar differential expression was not observed in the CPA of the validation cohort. The discrepancy might be explained by possible heterogeneity in CPA samples, speculated from previous adenoma studies (34). Decmann et al. recently reported heterogeneity associated with complex underlying pathology to be the possible reason for the observed discordance between NGS and qPCR of adenoma samples.

Finally, we also investigated the influence of short-term regulation of the pituitary-adrenal-axis on circulating miRNAs. For this analysis we focused on the 5 selected miRNAs miR-96-5p, miR-146b-5p, miR-182-5p, miR-183-5p, and miR-185-5p. Of the five selected miRNAs, miR-96-5p was significantly upregulated and miR-185-5p was significantly downregulated in post dexamethasone samples. Hormonal regulation of adrenal related miRNAs has been established in two murine *in vivo* studies for miR-96-5p (41, 42). For miR-185-5p, *in vitro* evidence of the influence of dexamethasone exist through the observed downregulation of the gene miR-185-5p during ameloblast differentiation under the influence of dexamethasone treatment (43). Therefore, it is tempting to speculate that the changes observed in the post dexamethasone samples could be a hint of possible hormonal regulation on the expression of these genes. Thusly, this influence needs to be taken into account in case of possible future application of the miRNAs in diagnostic setting involving dexamethasone.

The origin of circulating miRNAs is still subject to speculation. Since CS is not a malignant disease there is no concrete proof that the miRNAs significantly altered in the circulation are the result of specific tissue dysfunctions. Therefore, the study was limited only to identify and characterize circulating miRNA changes. Concurrently, there have been similar reports of increased miR182-5p expression in prolactin pituitary tumors (37) and ACTH induced up-regulation of miRNA-182-5p in murine adrenal glands. For a comprehensive mechanistic understanding behind 182-5p in CD in depth tissue based molecular analyses is required which is beyond the scope of the current paper (42).

In conclusion, we report distinct miRNA expression profiles associated with CD and CPA through NGS and qPCR confirmed miR-182-5p as the isolated miRNA to be differentially regulated. The identification of only one miRNA as a potential biomarker is speculated to be because of the following reasons: (1) We worked with 3 groups (CPA vs. CD vs. controls), and did an overall analysis and then post-hoc analysis. In this constellation it is more difficult to get significant results. (2) The number of patients per group was relatively low. (3) The controls were not healthy, but showed some similar clinical features as patients with Cushing’s syndrome. (4) As CPA and CD is a benign disease, there is no cell rupture and therefore, no release of miRNAs in the circulation. Technical and biological variations observed in the study warrants analysis using larger cohorts and more robust clinically relevant controls before considering miR-182-5p as a biomarker. Furthermore, our results suggest that presence or absence of ACTH might be at least as relevant for hypercortisolism per se.

## Data Availability Statement

The data sets presented in this study can be found in online repositories. The names of the repository/repositories and accession number(s) can be found below: https://www.ncbi.nlm.nih.gov/, GSE156693.

## Ethics Statement

The studies involving human participants were reviewed and approved by Ethics Committees of the University of Munich and Würzburg. The patients/participants provided their written informed consent to participate in this study.

## Author Contributions

SV, FB, MR, AC, and SS conceived and planned the experiments. BA, LB, AO, MB, MF, and MR collected the data and provided samples. SV, RZ, ME, and AR performed the experiments. FB, MR, AC, SS, and AR contributed to the interpretation of the results. SV and AR wrote the manuscript. All authors provided critical feedback. All authors contributed to the article and approved the submitted version.

## Funding

This work was supported by a grant from the Deutsche Forschungsgemeinschaft (DFG) (within the CRC/Transregio 205/1 “The Adrenal: Central Relay in Health and Disease”) to AO, MF, FB, MR, AC, and AR and individual grants SB 52/1-1 to SS and FA 466/5-1 to MF. This work is part of the German Cushing’s Registry CUSTODES and has been supported by grants from the Else Kröner-Fresenius Stiftung to MR (2012_A103 and 2015_A228) and SS **(**2016_A96). AR was supported by the FoeFoLe Program of the Ludwig-Maximilian-University Munich.

## Conflict of Interest

The authors declare that the research was conducted in the absence of any commercial or financial relationships that could be construed as a potential conflict of interest.
