# Supplementary material for: Circulating microRNA Expression in Cushing’s Syndrome
Source: Front Endocrinol (Lausanne). 2021 Feb 22;12:620012. doi: 10.3389/fendo.2021.620012 (PMC7937959; doi:10.3389/fendo.2021.620012)
Supplement: Supplementary file 1 [file DataSheet_1.pdf]

**Figure S1:** Analysis for differentially expressed miRNAs from NGS based on batch preparation (A), age (B) and sex (C). Volcano plot showing the relationship between fold change (log2foldchange) and statistical significance (-log10pvalue).

**Figure S2:** Differentially expressed miRNAs from NGS of postoperative group. Volcano plot showing the relationship between fold change (log2foldchange) and statistical significance (-log10pvalue). The red points in the plot represent the differentially expressed miRNAs with FDR <0.05 considered as statistically significant. CPA, cortisol producing adenoma; CD, Cushing's Disease, CS, Cushing's syndrome represents CPA and CD, taken together.

**Figure S3:** Results of QPCR of the significant miRNAs from the postoperative group analysis in the discovery cohort. Data are represented as Mean ± Standard deviation (SD) of -dCT values Statistics; ANOVA test with Bonferroni correction to detect significant differences between patient groups with at least a signification of p value <0.05.

**Figure S4:** Pie chart representing the top abundant genes from different patient groups. The top abundant genes were ranked based on their mean count from the miRNA sequencing data. No differences are observed in the top 25 most abundant miRNA amongst the groups.

**Table S1:**

Taqman probe IDs for QPCR analyses

| Gene | Taqman assay ID |
|---|---|
| miR-16-5p | 477860_mir |
| miR-96-5p | 478215_mir |
| miR-146b-5p | 483144_mir |
| miR-182-5p | 477935_mir |
| miR-183-5p | 477937_mir |
| miR-185-5p | 477939_mir |
| miR-342-3p | 478043_mir |
| miR-616-5p | 479099_mir |
| miR-629-5p | 478183_mir |
| miR-141-3p | 478501_mir |
| miR-200a-3p | 478490_mir |
| miR-215-5p | 478516_mir |
| mir-429 | 477849_mir |

**Table S2:**

**Correlation of miR-182-5p with clinical parameters**

| Group | baseline ACTH | Cortisol 24h Urine | midnight Cortisol | Cortisol after 1 mg Dexamethasone | BMI |
|---|---|---|---|---|---|
| **CS N=35** | 0.14 (0.42) | 0.18 (0.32) | -0.26 (0.18) | 0.02 (0.91) | -0.10 (0.58) |
| **CD N=19** | -0.04 (0.85) | -0.01 (0.95) | -0.09 (0.57) | 0.03 (0.54) | 0.03 (0.89) |
| **CPA N=16** | 0.27 (0.32) | 0.36 (0.19) | -0.48 (0.12) | 0.03 (0.92) | 0.10 (0.75) |

**Table S2:** The correlation of expression level of miR-182-5p (-Delta Ct) in QPCR with the clinical parameter of the Cushing patients from discovery and validation cohorts. All correlations were calculated using Spearman correlation procedure. Correlation coefficients (r) are given with the respective p values in brackets. $p < 0.05$ as considered significant. No significant correlations were observed. CPA, cortisol producing adenoma; CD, Cushing's Disease, CS, Cushing's syndrome represents CPA and CD, taken together.

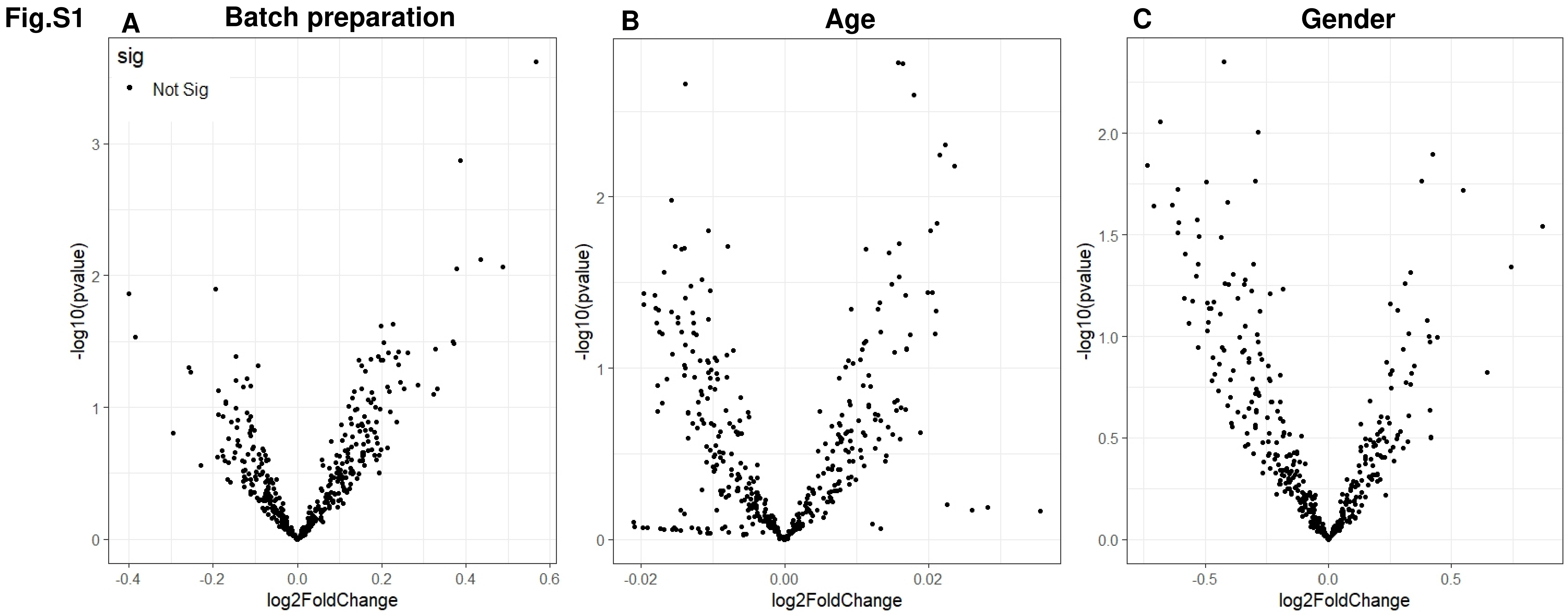

Fig.S1
A
Batch preparation
sig
Not Sig
-log10(pvalue)
log2FoldChange
B
Age
C
Gender

**Fig.S2**

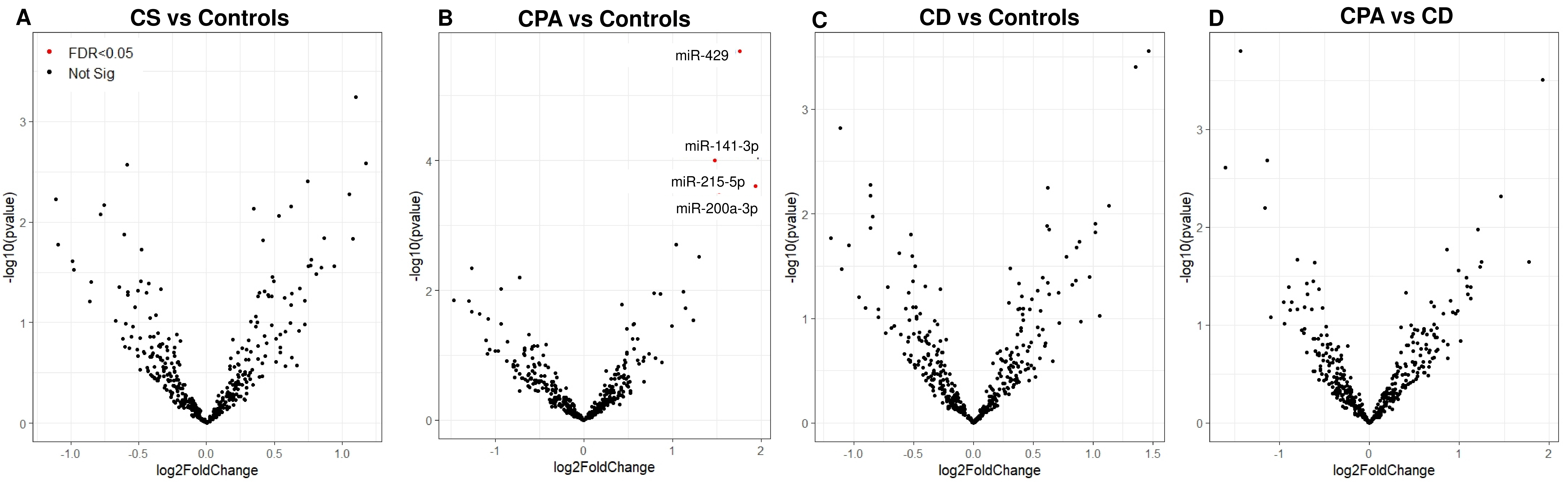
A
CS vs Controls
FDR<0.05
Not Sig
-log10(pvalue)
log2FoldChange
B
CPA vs Controls
miR-429
miR-141-3p
miR-215-5p
miR-200a-3p
C
CD vs Controls
D
CPA vs CD

Fig.S3

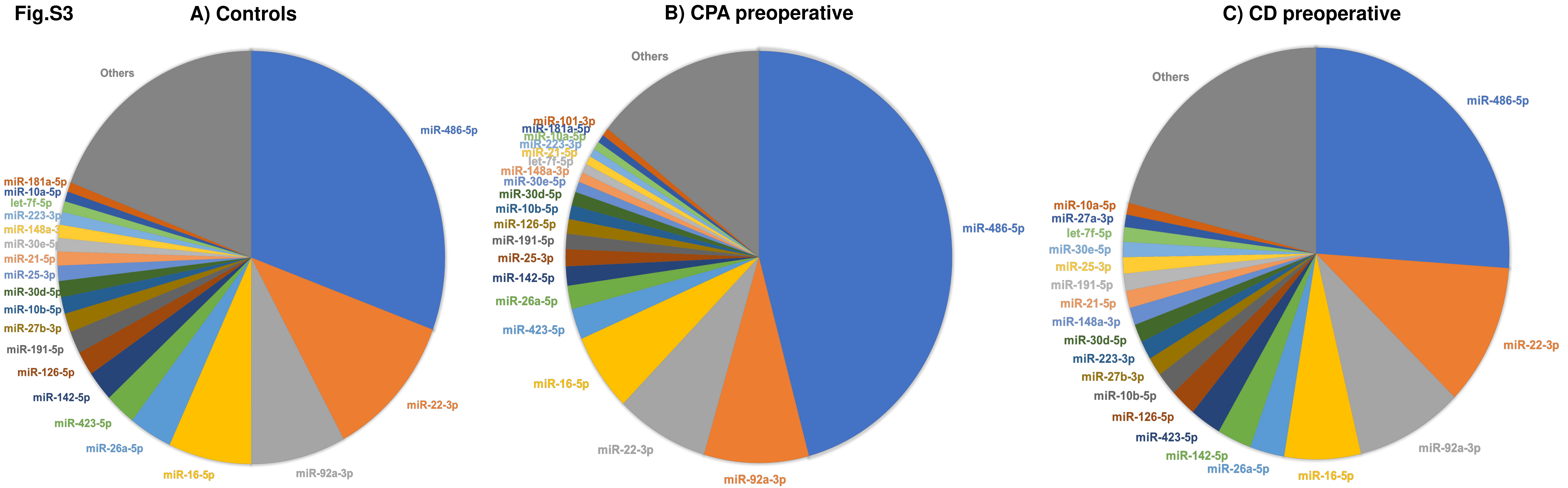

A) Controls
Others
miR-486-5p
miR-22-3p
miR-92a-3p
miR-16-5p
miR-26a-5p
miR-423-5p
miR-142-5p
miR-126-5p
miR-191-5p
miR-27b-3p
miR-10b-5p
miR-30d-5p
miR-25-3p
miR-21-5p
let-7f-5p
miR-10a-5p
miR-181a-5p
miR-223-3p
B) CPA preoperative
Others
miR-486-5p
miR-92a-3p
miR-22-3p
miR-16-5p
miR-423-5p
miR-26a-5p
miR-142-5p
miR-25-3p
miR-191-5p
miR-126-5p
miR-10b-5p
miR-30d-5p
miR-30e-5p
miR-148a-3p
let-7f-5p
miR-21-5p
miR-223-3p
miR-10a-5p
miR-181a-5p
miR-101-3p
C) CD preoperative
Others
miR-486-5p
miR-22-3p
miR-92a-3p
miR-16-5p
miR-26a-5p
miR-142-5p
miR-423-5p
miR-126-5p
miR-10b-5p
miR-27b-3p
miR-223-3p
miR-30d-5p
miR-148a-3p
miR-21-5p
miR-191-5p
miR-25-3p
miR-30e-5p
let-7f-5p
miR-27a-3p
miR-10a-5p

**Fig. S4**

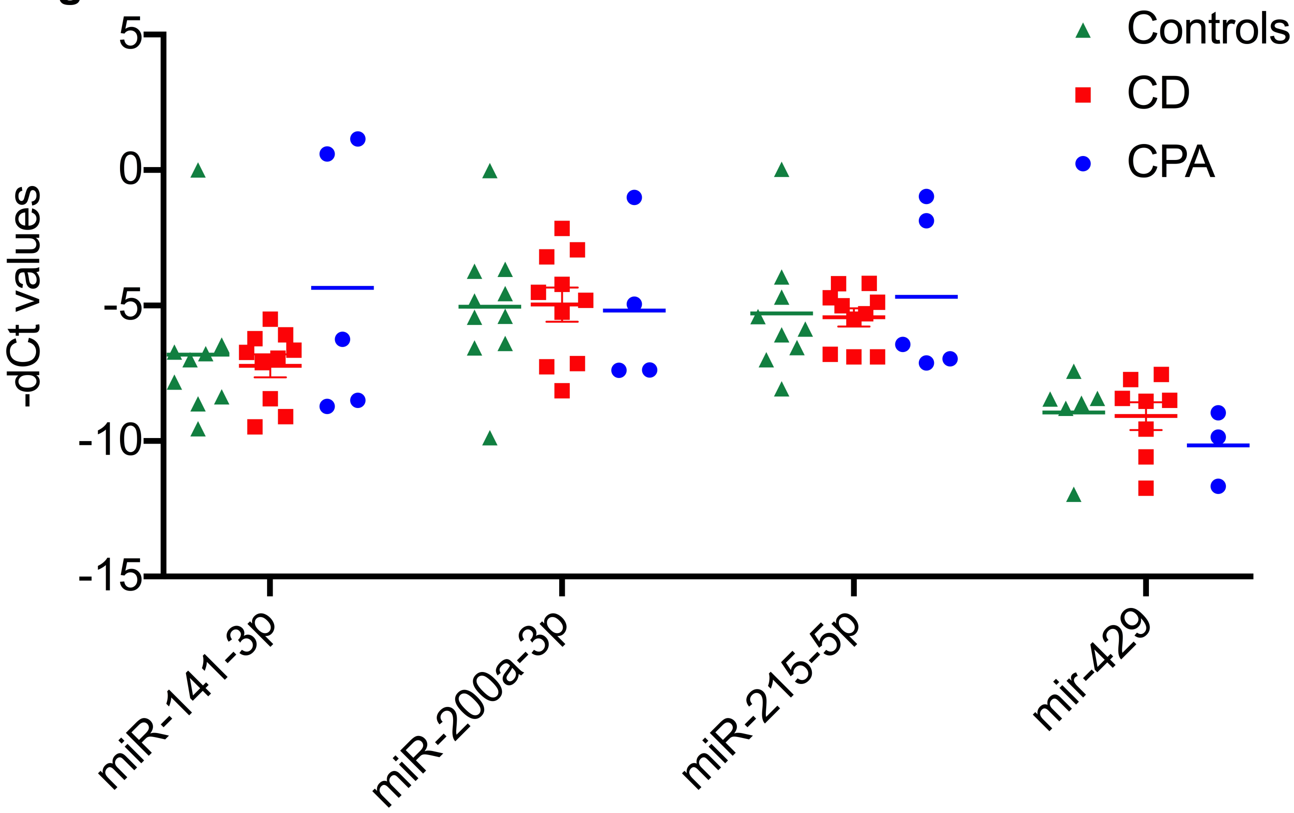

Controls
CD
CPA
5
0
-5
-10
-15
-dCt values
miR-141-3p
miR-200a-3p
miR-215-5p
mir-429
